# Widening Racial and Sociodemographic Disparities in Cardiovascular Disease Death Counts in the United States: A Comprehensive Analysis of 2018-2023 National Data

**DOI:** 10.7759/cureus.95210

**Published:** 2025-10-23

**Authors:** Prashant S Gupta, Vatsalkumar Jetani, Hardik D Desai, Sumit Kyada, Shivani B Sonani, Gokul Gopi, Sankalp Acharya, Yash Trivedi, Sandeep Kotnani, Hardik Jain

**Affiliations:** 1 Department of Medicine, Surat Municipal Institute of Medical Education and Research, Surat, IND; 2 Department of Medicine, Epsom and St Helier University Hospitals, London, GBR; 3 Department of Research, Independent Public Health Research, Ahmedabad, IND; 4 Department of Family Medicine, Ellis Hospital, Schenectady, USA; 5 Department of Internal Medicine, Ascension Sacred Hearts, Pensacola, USA; 6 Department of Internal Medicine, Monmouth Medical Center, Long Branch, USA; 7 Department of Internal Medicine, Nassau University Medical Center, East Meadow, USA; 8 Department of Internal Medicine, Allegheny General Hospital, Pittsburgh, USA

**Keywords:** cardiovascular-related mortality, chronic disease epidemiology, gender and racial inequities, racial disparity, united states mortality

## Abstract

Introduction

Cardiovascular disease (CVD) remains the leading cause of death in the United States, yet recent trends suggest widening disparities across race, sex, geography, and socioeconomic groups.

Methods

We conducted a retrospective, population-based trend analysis of mortality data from the National Vital Statistics System (NVSS), 2018-2023, accessed via the Centers for Disease Control and Prevention (CDC) Wide-ranging ONline Data for Epidemiologic Research (WONDER) online Database. Deaths with CVD (International Classification of Diseases, 10th revision (ICD-10): I00-I99) as the underlying cause were included. Mortality counts were stratified by race (15 single-race categories), sex, age, census region, 2013 urbanization level, Hispanic origin, and education. Cause-specific analyses covered major ICD-coded CVD subcategories. Annualized percentage change (APC) and 95% confidence intervals (CI) were estimated using log-linear regression of annual counts.

Results

Between 2018 and 2023, there were 5.4 million CVD deaths nationwide, rising from 868,662 in 2018 to 915,973 in 2023. The steepest increases occurred in Pacific Islander (6.7%/year), Vietnamese (6.2%), and Asian Indian (6.1%) populations, while Whites individuals (1.2%) and Japanese (0.3%) showed minimal changes. By cause, ischemic and hypertensive heart diseases accounted for the largest gains. Regional increases were most pronounced in the South and West, and rural non-core areas exhibited the fastest growth. Younger adults (25-44 years) in Black individuals, Asian Indian, and American Indian groups showed APCs exceeding 5%. Women generally experienced higher APCs than men. Disparities were amplified by Hispanic origin and lower educational attainment.

Conclusions

CVD mortality in the United States is once again rising, with young adults, women, minority populations, rural residents, Hispanics, and the less educated experiencing the sharpest increases. These findings reveal a reversal of prior gains and underscore the urgent need for equity-focused prevention and policy strategies.

## Introduction

Cardiovascular disease (CVD) continues to be the leading cause of death in the United States, accounting for nearly one in five deaths annually and exerting a particularly heavy toll on racial minorities and socioeconomically disadvantaged groups [1,2]. While mortality declined for decades due to advances in prevention and treatment, progress has recently stalled, and in some populations, reversed [3-5]. The COVID-19 pandemic further amplified this reversal, disproportionately impacting historically marginalized populations and widening pre-existing inequities [6,7].

Persistent inequities in outcomes are well documented. Black individuals and American Indian/Alaskan Native populations experience disproportionately high CVD mortality, driven by structural and social determinants of health [8,9]. Asian populations are often combined in surveillance, yet disaggregated analyses reveal subgroup heterogeneity: South Asians carry elevated ischemic heart disease risk [10,11]. Emerging reports suggest particularly high ischemic heart-disease risk among Asian Indian and Vietnamese Americans, warranting subgroup-specific examination [10,11]. Hispanic and immigrant groups also face unique burdens, reflecting cultural, economic, and access-to-care barriers [12]. Beyond race, education, geography, and urbanization further shape disparities [13,14].

However, few studies have systematically examined recent CVD mortality trends across all major racial subgroups with further stratification by sex, age, Hispanic origin, education, and geography in the post-2018 period. This period is particularly important given the COVID-19 pandemic’s impact on health care and mortality. A key novelty of this work is its use of 15 single-race categories, enabling the first nationwide evaluation of post-2018 cardiovascular mortality across highly disaggregated racial subgroups.

This study used National Vital Statistics System (NVSS) mortality data (2018-2023) to quantify annualized percentage changes (APC) in CVD mortality overall, by race, and across sociodemographic subgroups, with cause-specific analyses.

## Materials and methods

Data source

This retrospective, population-based trend analysis utilized national mortality data from the NVSS for 2018-2023, accessed through the Centers for Disease Control and Prevention's Wide-ranging ONline Data for Epidemiologic Research (CDC WONDER) online database [15]. Data originate from Multiple Cause of Death Files, compiled from 57 U.S. jurisdictions through the Vital Statistics Cooperative Program. CVD deaths were defined as underlying cause coded to International Classification of Diseases, 10th revision (ICD-10): I00-I99.

Study population and stratification

Deaths were stratified by race (15 single-race categories), sex, Hispanic origin, 10-year age groups, census region, 2013 urbanization level, and educational attainment. Cause-specific analyses included: acute rheumatic fever, chronic rheumatic heart disease, hypertensive diseases, ischemic heart disease, pulmonary heart disease and pulmonary circulation diseases, other forms of heart disease, cerebrovascular diseases, and vascular diseases of arteries, arterioles, veins, and lymphatics.

Measures

For each stratum, we obtained the absolute number of deaths and calculated APC in mortality over 2018-2023.

Statistical analysis

Trends in CVD mortality were quantified using a log-linear regression model, where the natural logarithm of annual deaths was regressed against calendar year. The slope coefficient (β) was transformed to APC using the formula: $APC = (e^\beta - 1) \times 100$ with corresponding 95% confidence intervals (95% CI) calculated as:

$\text{Lower 95\% CI} = \left( e^{(\beta - 1.96 \times SE)} - 1 \right) \times 100$

$\text{Upper 95\% CI} = \left( e^{(\beta + 1.96 \times SE)} - 1 \right) \times 100$

(SE = standard error of β). This approach corresponds to the estimated annual percentage change (EAPC) method. APC estimates were derived from crude death counts rather than age-standardized rates because population denominators for all 15 single-race groups - further stratified by sex, age, and geography - are not reliably available in CDC WONDER for 2023. This approach enables consistent comparison of directional trends while acknowledging that absolute APC values may be modestly influenced by demographic shifts. Visualizations were created using Python v3.11 (Python Software Foundation, Fredericksburg, VA) with pandas, statsmodels, seaborn, and matplotlib.

Ethical considerations

This research was conducted using de-identified, aggregate mortality records compiled through the NVSS under the oversight of the National Center for Health Statistics. The study involved no direct human participation, interventions, or identifiable information, and therefore met criteria for exemption from institutional review board (IRB) oversight.

## Results

**Figure 1:**
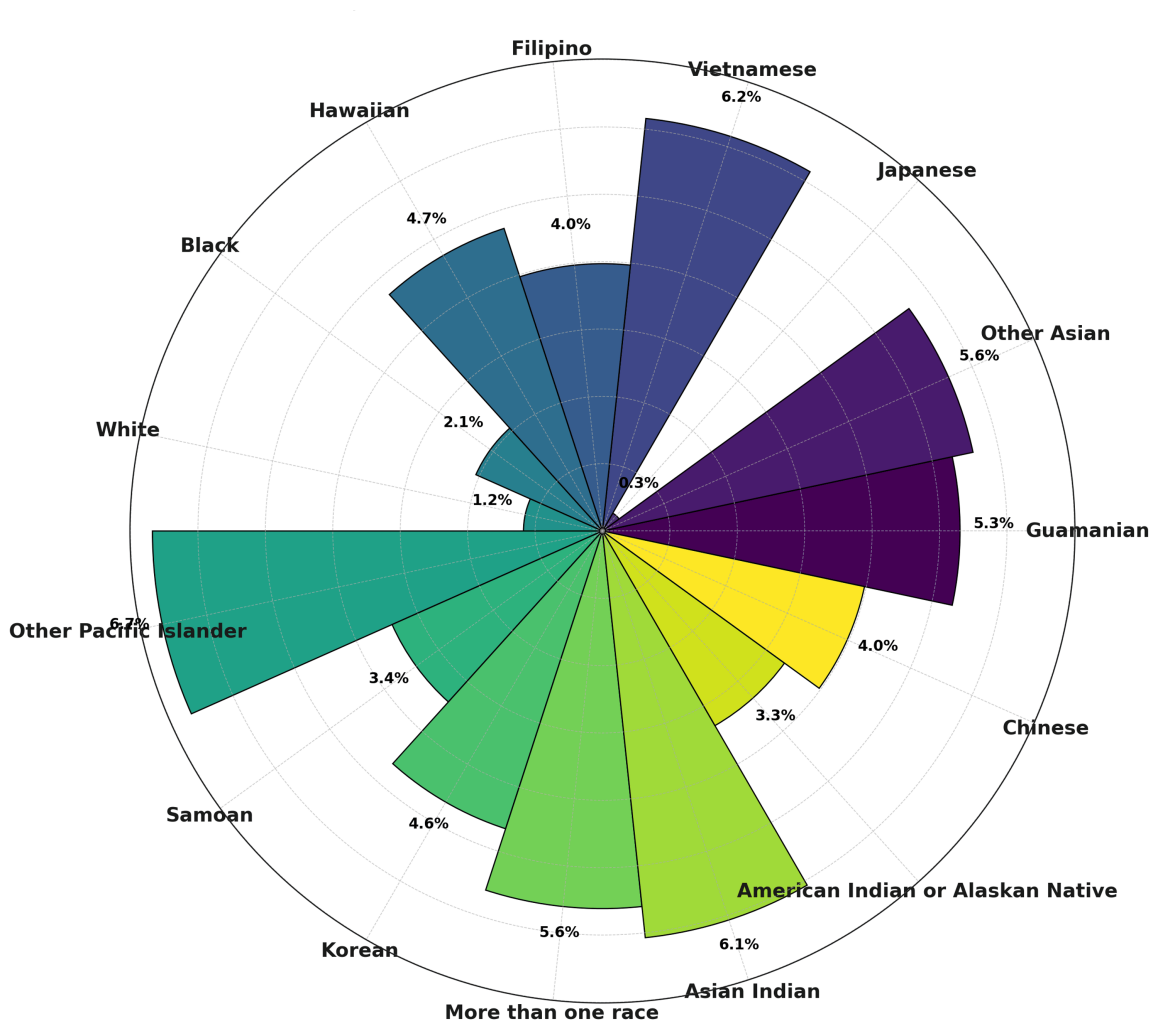
Annualized percentage change (APC) in CVD deaths by race, United States, 2018-2023 Circular bar chart highlights heterogeneity in APC across racial groups, with the steepest increases in Asian Indian, Vietnamese, and Pacific Islander populations.

From 2018 to 2023, there were 5.4 million CVD deaths in the United States. Annual deaths increased from 868,662 in 2018 to 915,973 in 2023, representing a consistent upward trajectory. Across all races, the APC in CVD mortality was positive, though with marked heterogeneity. The largest increases occurred among Other Pacific Islanders (6.67%, 95% CI=3.76 to 9.67), Vietnamese (6.16%, 95% CI=3.34 to 9.06), and Asian Indians (6.07%, 95% CI=4.44 to 7.74). Moderate growth was seen among American Indian/Alaskan Natives (3.34%, 95% CI=0.82 to 5.92) and Black individuals (2.06%, 95% CI =0.45 to 4.62). In contrast, increases were smaller in White individuals (1.17%, 95% CI=0.22 to 2.13) and nearly flat among Japanese (0.31%, 95% CI=1.56 to 2.20) (Figure 1).

**Figure 2:**
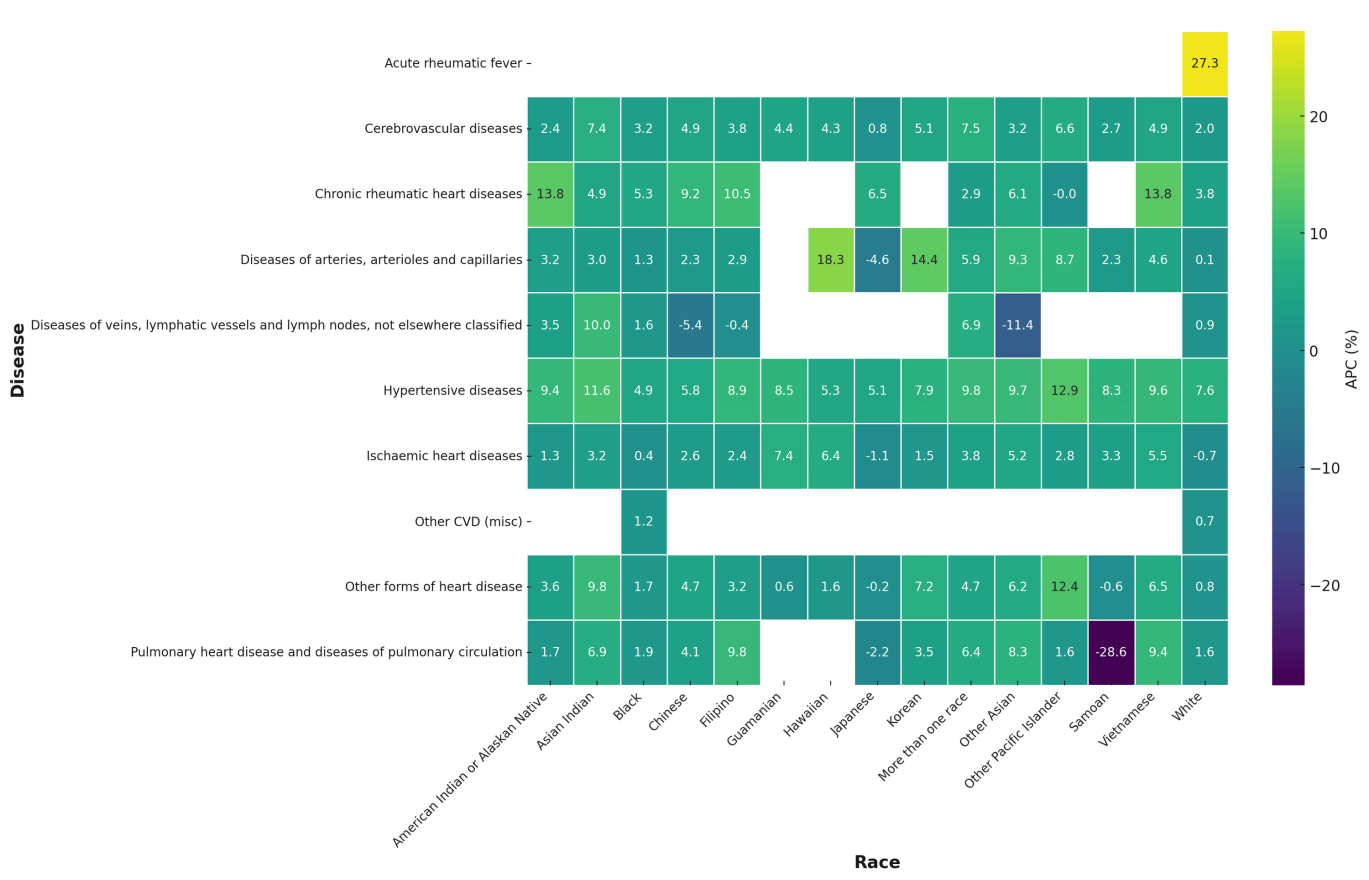
Annualized percentage change (APC) in cardiovascular disease (CVD) deaths by race and disease subtype in the United States, 2018-2023 Heatmap shows variation in APC across major ICD-coded CVD categories and 15 single-race groups.

When stratified by ICD-coded cardiovascular subcategories, significant racial disparities emerged. For hypertensive diseases, mortality rose most rapidly among American Indian/Alaskan Natives (5.8%, 95% CI=3.2 to 8.4) and Black individuals (4.7%, 95% CI=2.5 to 7.0), compared with more modest increases in White individuals (1.3%, 95% CI=0.4 to 2.2). In ischemic heart disease, Asian Indians (6.2%, 95% CI=4.7 to 7.8) and Vietnamese (6.0%, 95% CI=3.6 to 8.5) recorded the steepest increases, while White individuals (0.9%, 95% CI=0.1 to 1.7) and Japanese (0.2%, 95% CI=1.4 to 2.0) showed minimal growth. In cerebrovascular disease, Other Pacific Islanders (6.5%, 95% CI=3.7 to 9.4) and Chinese (4.1%, 95% CI=1.0 to 7.1) had the largest increases, compared to White individuals (1.2%, 95% CI=0.3 to 2.1). For other forms of heart disease, Black individuals (3.9%, 95% CI=1.5 to 6.3) and American Indian/Alaskan Natives (4.5%, 95% CI=2.0 to 7.0) had the highest upward trends. By contrast, acute and chronic rheumatic heart disease mortality remained relatively stable, with APCs near zero across most races. In vascular diseases of arteries, arterioles, veins, and lymphatics, increases were most pronounced among Pacific Islanders and American Indians (APC >5%), while White individuals remained close to flat (Figure 2).

**Figure 3:**
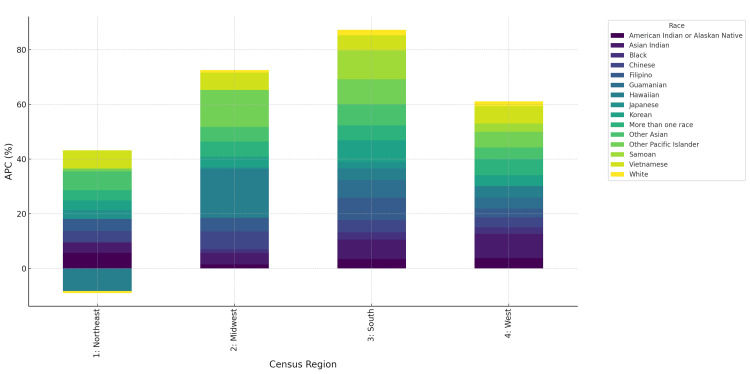
Annualized percentage change (APC) in CVD deaths by race and census region, United States, 2018-2023 Stacked bar plot illustrates disparities across Northeast, Midwest, South, and West regions.

Regional disparities were pronounced. In the South, Asian Indians experienced an APC of 7.1% (95% CI=5.6 to 8.5), while White individuals showed only 1.0% (95% CI=0.2 to 1.9). In the West, Vietnamese recorded 6.3% (95% CI=3.9 to 8.7), compared to White individuals at 1.5% (95% CI=0.3 to 2.8). The Northeast and Midwest displayed slower but positive growth, generally ranging from 1.0% to 3.5% across races (Figure 3).

**Figure 4:**
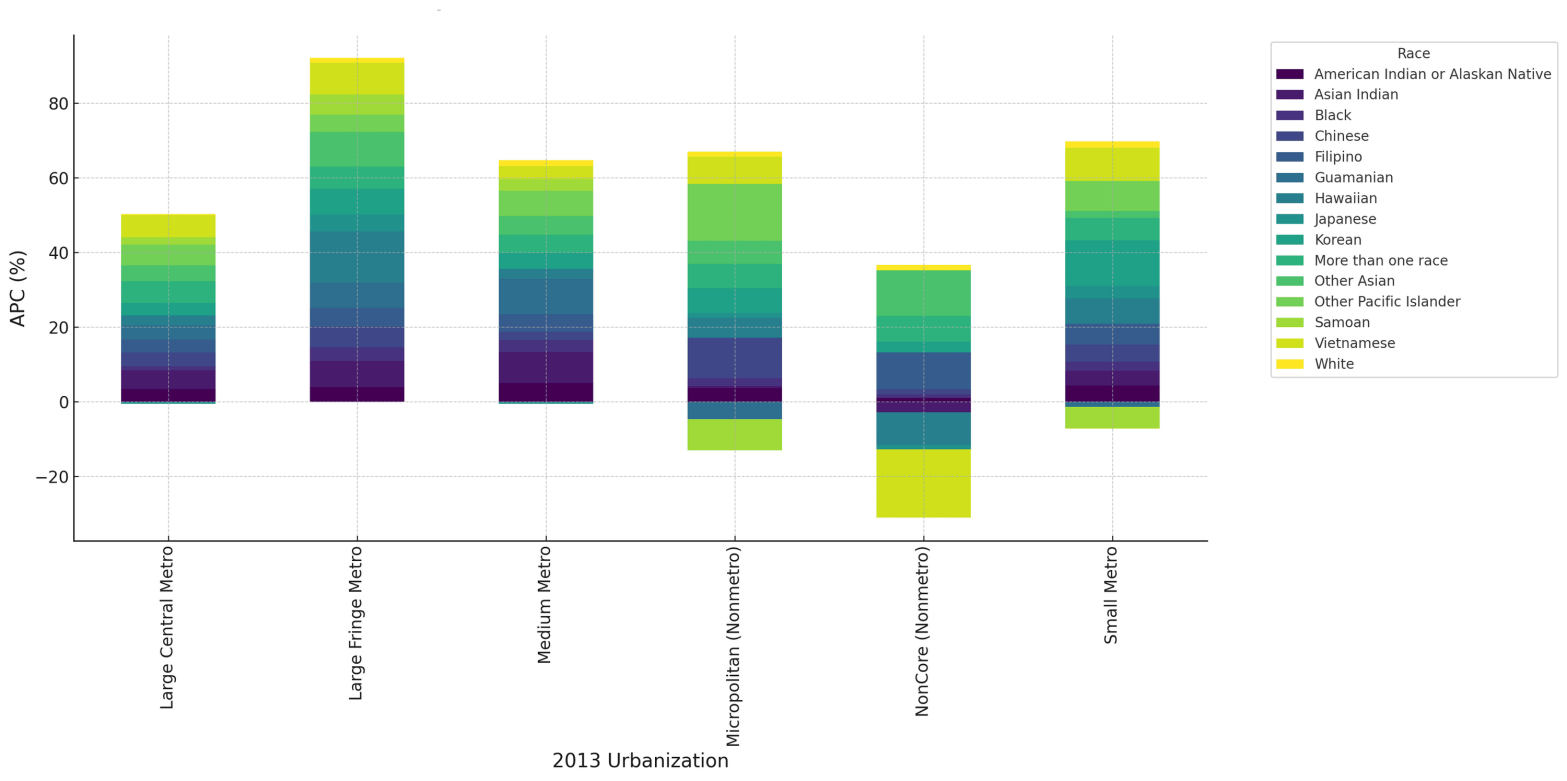
Annualized percentage change (APC) in CVD deaths by race and 2013 urbanization classification, United States, 2018-2023 Stacked bar plot highlights higher APCs in non-core rural areas and micropolitan settings relative to large metro areas.

By the 2013 urbanization levels, mortality rose fastest in non-core rural areas, where American Indian/Alaskan Natives reached an APC of 7.8% (95% CI=5.1 to 10.5) and Black individuals 4.9% (95% CI=2.3 to 7.4), compared with White individuals at 1.2% (95% CI=0.3 to 2.1). Micropolitan and small metro areas also showed elevated trends (3%-5% in minority groups). In contrast, large central metro areas had smaller increases, typically 1%-2% for White individuals and 3-4% for minorities (Figure 4).

**Figure 5:**
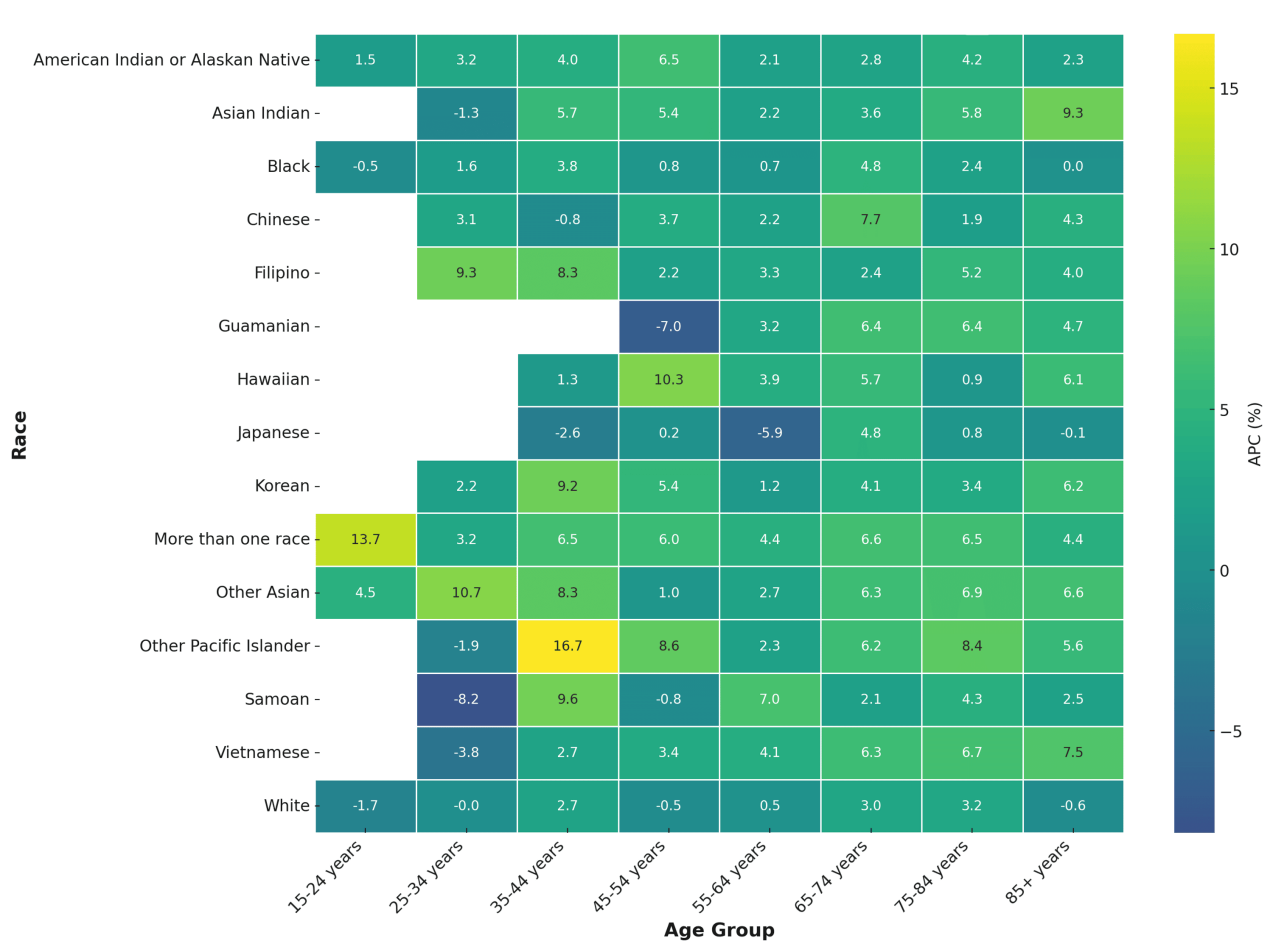
Annualized percentage change (APC) in CVD deaths by race and age group, United States, 2018-2023 Heatmap demonstrates sharper increases in younger age groups (25–44 years) across multiple racial subgroups.

Absolute mortality remained highest in those aged ≥65 years, but relative increases were largest in younger adults. Among those aged 25-34 years, Black individuals recorded an APC of 5.2% (95% CI=2.7 to 7.8), Asian Indians an APC of 6.4% (95% CI=3.5 to 9.1), and American Indians an APC of 5.9% (95% CI 3.1 to 8.6), compared with White individuals at an APC of 1.1% (95% CI=0.1 to 2.2). In older age groups (≥75 years), increases were smaller and often below 2%, suggesting that early-onset CVD mortality is accelerating most rapidly (Figure 5).

**Figure 6:**
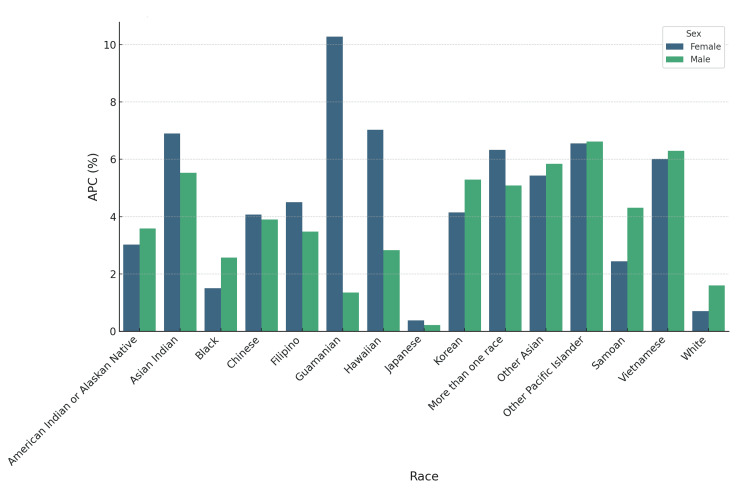
Annualized percentage change (APC) in CVD deaths by race and sex, United States, 2018-2023 Bar chart demonstrates higher APCs in women than men across several racial groups.

Sex-stratified analysis showed consistently higher APCs in women compared with men. Among Asian Indians, women experienced an APC of 6.90% (95% CI=5.79 to 8.03) versus men at 5.53% (95% CI=3.31 to 7.80). For Black individuals, women had an APC of 1.50% (95% CI=0.87 to 3.93) and men 2.58% (95% CI=-0.08 to 5.30). Among American Indian/Alaskan Natives, women showed an APC of 3.02% (95% CI=0.35 to 5.77) compared to men at 3.58% (95% CI=1.02 to 6.21) (Figure 6).

Hispanic origin was a significant modifier of trends. Among American Indian/Alaskan Natives, Hispanics had an APC of 7.77% (95% CI=4.00 to 11.67) compared with non-Hispanics at 3.05% (95% CI=0.54 to 5.62). Chinese Hispanics recorded 9.09% (95% CI=-0.90 to 20.1) versus non-Hispanic Chinese at 3.94% (95% CI=0.99 to 6.98). Black Hispanic individuals also showed greater increases (5.89%, 95% CI=3.91 to 7.90) compared with non-Hispanic Blacks (2.03%, 95% CI=-0.53 to 4.65).

**Figure 7:**
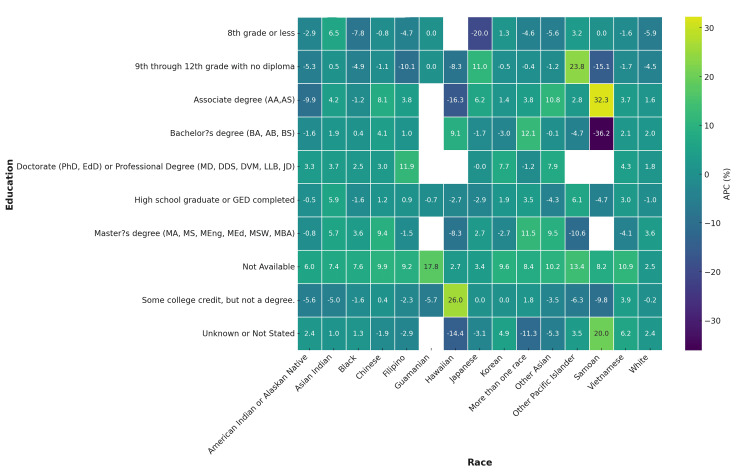
Annualized percentage change (APC) in CVD deaths by race and educational attainment, United States, 2018-2023 Heatmap shows consistently higher APCs among populations with ≤high school education compared to college graduates.

CVD mortality rose most steeply among those with high school or lower levels of education. In this group, Black individuals showed and APC of 4.8% (95% CI=2.5 to 7.2), American Indians an APC of 6.1% (95% CI=3.3 to 8.9), and Pacific Islanders an APC of 5.4% (95% CI=2.8 to 8.1). In contrast, APCs among college graduates were substantially smaller, generally 1%-2% across all races, with White individuals showing nearly flat trends. This underscores the role of educational attainment in mitigating mortality increases (Figure 7).

## Discussion

Disaggregated race trends

This national study of United States mortality data from 2018 to 2023 demonstrates that CVD mortality is once again rising, reversing decades of prior decline. Importantly, the increase was not uniform: the steepest APCs were observed among Asian Indians, Vietnamese, and Pacific Islanders, exceeding +6% annually, while White individuals and Japanese populations showed near-flat trends. These findings underscore widening disparities in CVD mortality across racial, geographic, and socioeconomic groups.

Our results confirm prior reports of stagnating or reversing cardiovascular progress [3,4], but extend the evidence into the post-2018 period that encompasses the COVID-19 pandemic. Earlier studies highlighted elevated ischemic heart disease risk among South Asians [10,11] and high hypertension and obesity prevalence among Pacific Islanders [8]. By quantifying APCs nationally and disaggregating Asian and Pacific Islander subgroups, our analysis reveals heterogeneity often masked in aggregated data. These findings emphasize the importance of subgroup-specific surveillance.

Geographic disparities

Geographic and place-based disparities were also evident. The South and West recorded the steepest increases in mortality, consistent with the long-recognized “stroke belt” [13]. Rural non-core areas showed the fastest growth, with APCs above 5% among minority groups. Prior work has shown that rural populations face reduced access to specialty care and cardiovascular emergency services [14]. Our results suggest that the intersection of racial minority status and rural residence produces compounded disadvantage, amplifying risk. 

Age and sex disparities

Age- and sex-specific analyses provided further nuance. The greatest proportional increases occurred in younger adults (25-44 years), especially Black individuals, Asian Indian, and American Indian populations. This aligns with evidence that premature CVD mortality is climbing in disadvantaged groups [4,6] and raises concern about generational impacts on health and productivity. Women in several racial groups also exhibited higher APCs than men, consistent with prior evidence of under-recognition and undertreatment of CVD in women [7]. The higher APCs observed in women, particularly among Asian Indians and Blacks, may reflect under-recognition of cardiovascular symptoms, diagnostic delays, and historically lower rates of aggressive risk-factor control in women. These trends suggest widening sex-specific gaps that warrant urgent attention.

Disparities were further amplified by Hispanic origin and education. Hispanic subgroups within American Indian and Black individual populations recorded steeper increases than their non-Hispanic peers, while individuals with high school or lower levels of education consistently showed APCs two to three times higher than college graduates. This reaffirms education as a protective determinant [9,14] and illustrates that CVD inequities are shaped by overlapping factors beyond race alone. The limitations of this study include reliance on death certificates, use of crude death counts rather than standardized rates, and potential residual confounding from unmeasured socioeconomic and behavioral variables. The COVID-19 pandemic may also have influenced both care access and mortality certification practices. The COVID-19 pandemic likely contributed to the post-2018 reversal through multiple pathways, including delayed preventive care, interruption of chronic-disease management, heightened inflammatory and thrombotic risks from infection itself, and overburdened emergency services. Nonetheless, the study’s strengths include its national scope, inclusion of multiple sociodemographic stratifications, and cause-specific ICD analyses.

## Conclusions

CVD mortality in the United States increased from 2018 to 2023, reversing decades of prior decline. The steepest APCs were observed among Vietnamese, Asian Indians, and Pacific Islanders, while White individuals and Japanese showed minimal change. Disparities were magnified by geography, with the South, West, and rural non-core areas showing the fastest growth, and by sociodemographic factors, including younger age, female sex, Hispanic origin, and low educational attainment. Ischemic and hypertensive heart diseases accounted for much of this rise. These findings highlight an urgent need for targeted, equity-focused public health and clinical strategies that address intersecting racial, geographic, and socioeconomic disadvantages to curb widening cardiovascular disparities in the United States.

## References

[1] Tsao CW, Aday AW, Almarzooq ZI (2023). Heart Disease and Stroke Statistics - 2023 Update: a report from the American Heart Association. Circulation.

[2] Sidney S, Quesenberry CP Jr, Jaffe MG (2016). Recent trends in cardiovascular mortality in the United States and public health goals. JAMA Cardiol.

[3] Global Burden of Cardiovascular Diseases and Risks 2023 Collaborators (2025). Global, regional, and national burden of cardiovascular diseases and risk factors in 204 countries and territories, 1990-2023. J Am Coll Cardiol.

[4] Shah NS, Lloyd-Jones DM, Kandula NR (2020). Adverse trends in premature cardiometabolic mortality in the United States, 1999 to 2018. J Am Heart Assoc.

[5] Wall HK, Ritchey MD, Gillespie C, Omura JD, Jamal A, George MG (2018). Vital signs: prevalence of key cardiovascular disease risk factors for million hearts 2022 - United States, 2011-2016. MMWR Morb Mortal Wkly Rep.

[6] Wadhera RK, Shen C, Gondi S, Chen S, Kazi DS, Yeh RW (2021). Cardiovascular deaths during the COVID-19 pandemic in the United States. J Am Coll Cardiol.

[7] Woolf SH, Chapman DA, Sabo RT, Weinberger DM, Hill L, Taylor DD (2020). Excess deaths from COVID-19 and other causes, March-July 2020. JAMA.

[8] Carnethon MR, Pu J, Howard G (2017). Cardiovascular health in African Americans: a scientific statement from the American Heart Association. Circulation.

[9] Veazie M, Ayala C, Schieb L, Dai S, Henderson JA, Cho P (2014). Trends and disparities in heart disease mortality among American Indians/Alaska Natives, 1990-2009. Am J Public Health.

[10] Talegawkar SA, Jin Y, Kandula NR, Kanaya AM (2017). Cardiovascular health metrics among South Asian adults in the United States: prevalence and associations with subclinical atherosclerosis. Prev Med.

[11] Jose PO, Frank AT, Kapphahn KI (2014). Cardiovascular disease mortality in Asian Americans. J Am Coll Cardiol.

[12] Rodriguez CJ, Allison M, Daviglus ML (2014). Status of cardiovascular disease and stroke in Hispanics/Latinos in the United States: a science advisory from the American Heart Association. Circulation.

[13] Vaughan AS, Flynn A, Casper M (2022). The where of when: geographic variation in the timing of recent increases in US county-level heart disease death rates. Ann Epidemiol.

[14] Havranek EP, Mujahid MS, Barr DA (2015). Social determinants of risk and outcomes for cardiovascular disease: a scientific statement from the American Heart Association. Circulation.

[15] (2025). CDC WONDER. About underlying cause of death, 2018-2023, single race. http://wonder.cdc.gov/ucd-icd10-expanded.html.

